# Supplementary material for: Association between oral microbiome and seven types of cancers in East Asian population: a two-sample Mendelian randomization analysis
Source: Front Mol Biosci. 2023 Nov 21;10:1327893. doi: 10.3389/fmolb.2023.1327893 (PMC10702768; doi:10.3389/fmolb.2023.1327893)
Supplement: Supplementary file 1 [file DataSheet1.ZIP › Supplementary Materials/MR plots for tongue/tongue═╝/Ovarian cancer/pheno.2959_to_ovarian cancer_forest.pdf]

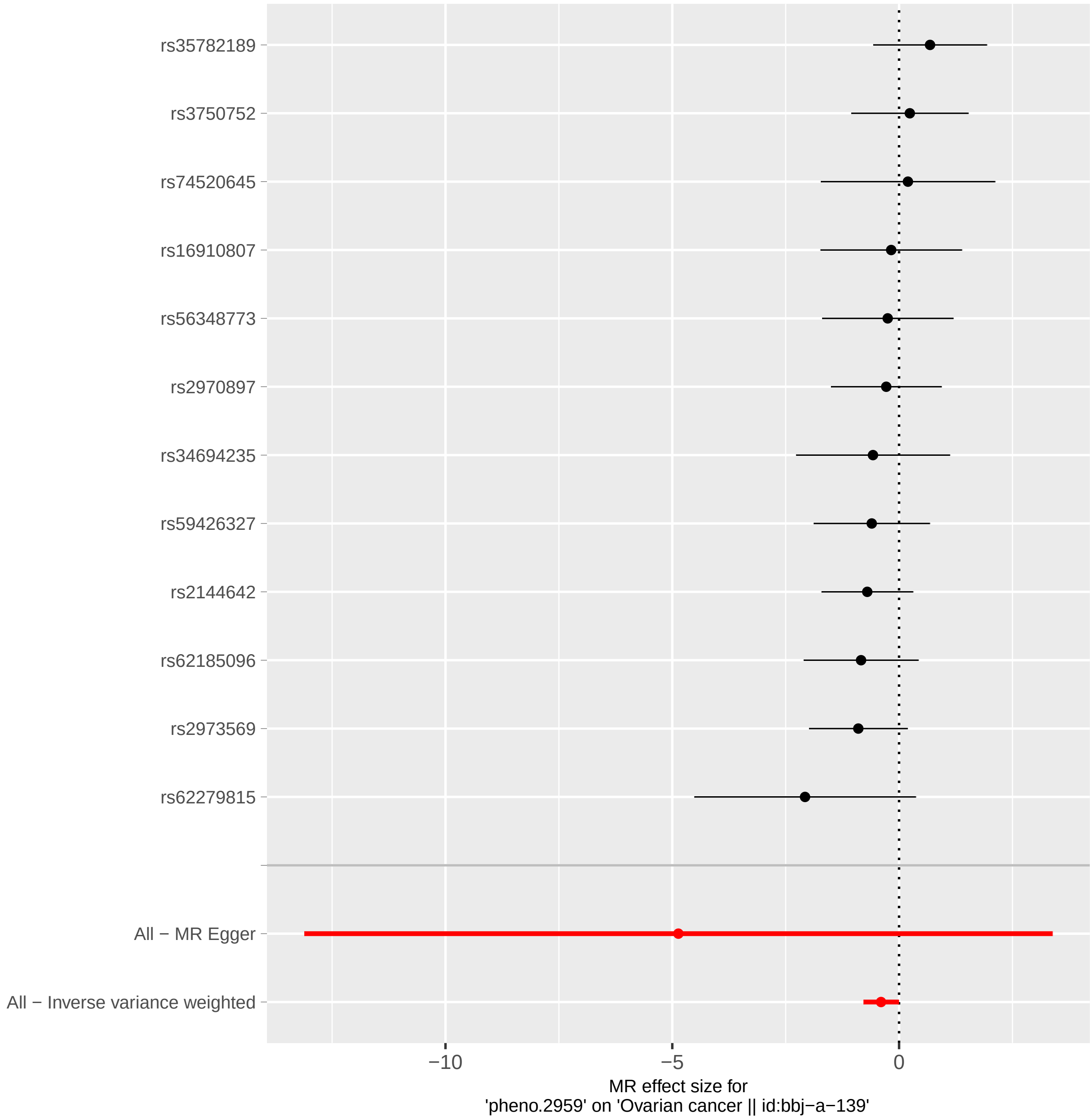
rs35782189
rs3750752
rs74520645
rs16910807
rs56348773
rs2970897
rs34694235
rs59426327
rs2144642
rs62185096
rs2973569
rs62279815
All − MR Egger
All − Inverse variance weighted
−10
−5
0
MR effect size for
'pheno.2959' on 'Ovarian cancer || id:bbj−a−139'
